# Secular Trends in the Prevalence of and Disability-Adjusted Life Years Due to Common Micronutrient Deficiencies in China From 1990 to 2019: An Age-Period-Cohort Study and Joinpoint Analysis

**DOI:** 10.3389/fnut.2022.754351

**Published:** 2022-03-18

**Authors:** Haiying Chen, Jinxin Lu, Yongze Li

**Affiliations:** ^1^Department of Obstetrics, The First Hospital of China Medical University, Shenyang, China; ^2^Department of Clinical Nutrition, The First Hospital of China Medical University, Shenyang, China; ^3^Department of Endocrinology and Metabolism and the Institute of Endocrinology, The First Hospital of China Medical University, Shenyang, China

**Keywords:** micronutrient deficiency, iodine deficiency, vitamin A deficiency, dietary iron deficiency, epidemiology

## Abstract

**Background:**

Understanding the national burdens and trends of micronutrient deficiencies can help guide effective intervention strategies. However, there is a lack of evidence of secular trends and age and sex differences in China. This study aims to elucidate trends in common micronutrient deficiencies, in particular, dietary iron, iodine and vitamin A deficiencies in China, from 1990 to 2019 using Global Burden of Disease (GBD) 2019 study data.

**Methods:**

Prevalence and DALYs trends of common micronutrient deficiencies from 1990 to 2019 were assessed by joinpoint regression analysis. Age, period and cohort effects on the prevalence of common micronutrient deficiencies were estimated by an age-period-cohort model.

**Results:**

From 1990 to 2019, the age-standardized prevalence rates of iodine, vitamin A and dietary iron deficiencies changed by −0.6% (95% CI: −0.7% to −0.5%), −6.3% (−6.6% to −6.0%), and −3.5% (−3.6% to −3.4%) in males and + 0.8% (+ 0.6% to + 1.0%), −4.5% (−4.8% to −4.2%), and −3.3% (−3.4% to −3.2%) in females, respectively. The average annual percent change (AAPC) in the iodine deficiency prevalence increased in females aged 20 years and older. The relative risk (RR) of iodine deficiency associated with the age effect peaked at 30–34 years of age and then decreased with increasing age. The RR of vitamin A deficiency decreased with age. The age distribution of the RR of iron deficiency differed significantly between sexes. The RRs of vitamin A deficiency and dietary iron deficiency decreased over time, whereas the RR of iodine deficiency substantially increased starting in 2004. The RRs of iodine deficiency and dietary iron deficiency associated with the cohort effect decreased, but the vitamin A deficiency prevalence increased in successive birth cohorts.

**Conclusion:**

Micronutrient deficiency prevalence rates and associated DALYs decreased from 1990 to 2019 in China. Young adults, children aged less than 5 years, and older individuals were disproportionately affected by iodine, vitamin A, and dietary iron deficiencies, respectively. The results of this study may help identify individuals who would benefit from interventions to improve micronutrient deficiency.

## Introduction

Micronutrient deficiency is an important contributor to the global burden of disease because it contributes to increases in incidence and mortality. An estimated 2 billion individuals worldwide suffer from at least one form of micronutrient deficiency (1). This burden exists worldwide, especially affecting children and pregnant women. According to a report from the World Health Organization (WHO), iodine, vitamin A and iron deficiencies of are of great concern worldwide (2).

Iodine deficiency in children leads to intellectual impairment and growth retardation and is an important cause of preventable mental disorders worldwide (2). China was previously identified as a severely iodine-deficient country; thus, mandatory universal salt iodization was introduced in 1996 (3). Vitamin A deficiency is associated with night blindness, impaired immune function and increases mortality due to measles and diarrhea among children (4, 5). It is estimated that approximately 10% of preschool children have been affected by vitamin A deficiency, which indicates that vitamin A deficiency has public health significance in China (6). In addition, individuals with iron deficiency may experience symptoms such as muscle weakness, dizziness, shortness of breath, fatigue and impaired immune function (7). Iron-deficiency anemia is the leading contributor to the burdens of maternal, neonatal and nutritional disorders in China according to the Global Burden of Disease (GBD) study 2010 (8).

Most deficiencies can be prevented through health education and the implementation of strategies promoting a healthy diet containing diverse foods, as well as fortified and supplemented foods, such as universal salt iodization (USI), vitamin A supplementation in children, and iron/folate supplementation in pregnant women. Assessing the current burden and secular trends of micronutrient deficiencies are critical to understanding their status under various conditions and evolving interventions. Previous studies on micronutrient deficiencies have been mainly focused on subpopulations or limited to certain regions in China (9, 10). In addition, sociodemographic development, economic transformation, and risk exposure have varied substantially over the past three decades. Therefore, the burden of micronutrient deficiency has also changed accordingly.

Given the limited epidemiological information on micronutrient deficiency in the Chinese population, more updated estimations are needed to help guide future research on disease control and prevention strategies. Recently, the GBD and Injuries Collaborators reported the global burdens of 369 diseases and injuries in 204 countries and territories from 1990 to 2019 (11, 12). Therefore, this study analyzed data from the GBD 2019 to examine the temporal trends in the prevalence of and disability-adjusted life years (DALYs) due to dietary iron deficiency, iodine deficiency, and vitamin A deficiency in China and to explore the net age, period, and cohort effects within the age-period-cohort framework.

## Materials and Methods

### Data Sources

Data on micronutrient deficiency burdens in China from 1990 to 2019 were obtained from the Global Health Data Exchange GBD Results Tool^1^ (date of data extraction, May 20, 2021). The GBD 2019 incorporated all available epidemiological data and improved standardized methods, taking into account 369 diseases and injuries and 87 risk factors in 204 countries and regions, and conducted a comprehensive assessment of health loss (11, 12). Previous studies have described the general approach to the GBD 2019, including major changes compared to the previous year (11, 12). In brief, the GBD 2019 used standardized tools to model processed data to estimate each variable of interest based on age, sex, location, and year. The GBD 2019 used various related indicators to measure population health loss, including the number of deaths and mortality, the number of cases and prevalence, years of life lost (YLLs) due to premature death, years of life lived with disability (YLDs), and DALYs. In this study, we used the GBD results tool to extract estimates of the prevalence and DALYs and their 95% uncertainty intervals in China from 1990 to 2019 to measure the burden of dietary iron deficiency, iodine deficiency, and vitamin A deficiency.

### Case Definitions

The assessment of the burden of vitamin A deficiency included the quantification of total vitamin A deficiency (serum retinol <0.7 μmol/L), as well as blindness and vision loss caused by vitamin A deficiency, which are related to corneal ulcers and corneal scars (11). Dietary iron deficiency was defined as insufficient dietary iron that cannot meet the body’s needs due to insufficient dietary iron intake rather than due to other absolute or functional iron deficiencies (11). The non-fatal burden of iodine deficiency included iodine deficiency associated with visible goiter (grade 2) and its related sequelae, including thyroid dysfunction, heart failure, and intellectual disability. It did not include estimates of subclinical iodine deficiency or invisible goiter (grade 1) caused by iodine deficiency (11).

### Data Analysis

The joinpoint regression model is a set of linear statistical models, which was used for assessing the trends of disease burdens due to micronutrient deficiencies over time. The calculation principle of this model is to use the least square method to estimate the changing rule of disease rates, thereby avoiding the unobjectivity of traditional trend analysis based on linear trends. The turning point of the changing trend is determined by calculating the square sum of the residual error between the estimated value and the actual value. This model was performed by the Joinpoint software (version 4.7). In addition, the average annual percentage change (AAPC) was calculated. By comparing AAPC with 0, we determined whether the variation trend in different sections was statistically significant. Furthermore, the age-period-cohort model is developed to reflect the relative risk (RR) of micronutrient deficiency prevalence rates by estimating the age, period, and cohort effects. In the age-period-cohort model, individuals were divided into consecutive 5-year age groups (0–4, 5–9,…, 70–74, 75–79), consecutive 5-year periods (1994, 1999, 2004, 2009, 2014, and 2019), and corresponding consecutive 5-year birth cohort groups (1915–1919, 1920–1924, …, 2010–2014, and 2015–2019). The exponential value of the estimated coefficients of age, period, and cohort effects indicates the RR of age, period, and cohort effect. This model was conducted using STATA software (version 14.0).

## Results

**FIGURE 1:**
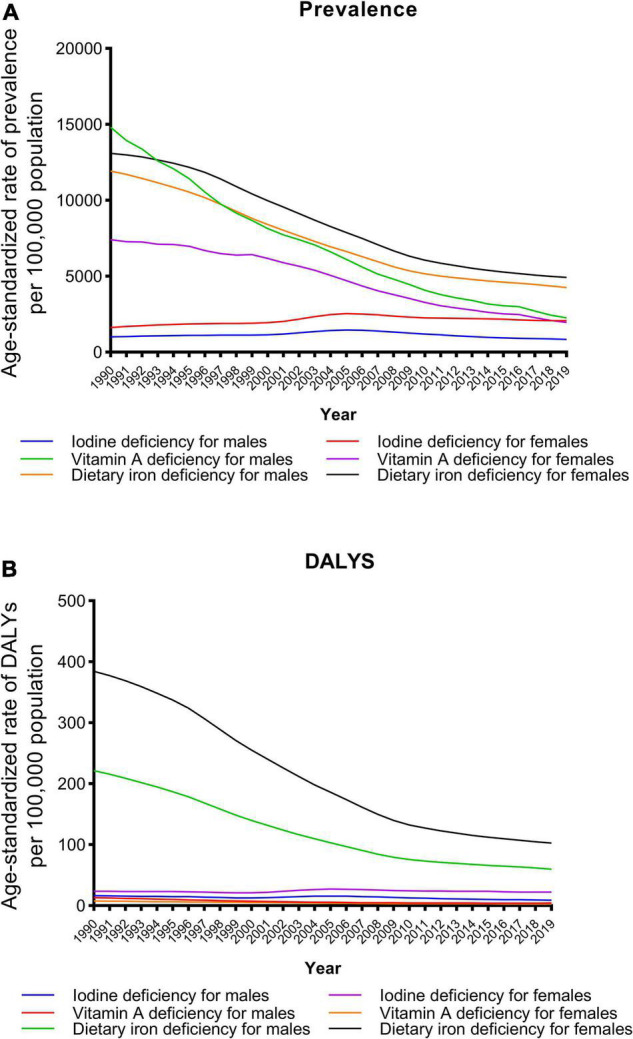
Trends in the age-standardized prevalence **(A)** and DALY **(B)** rates of iodine deficiency, vitamin A deficiency and dietary iron deficiency by sex from 1990 to 2019.

**FIGURE 2:**
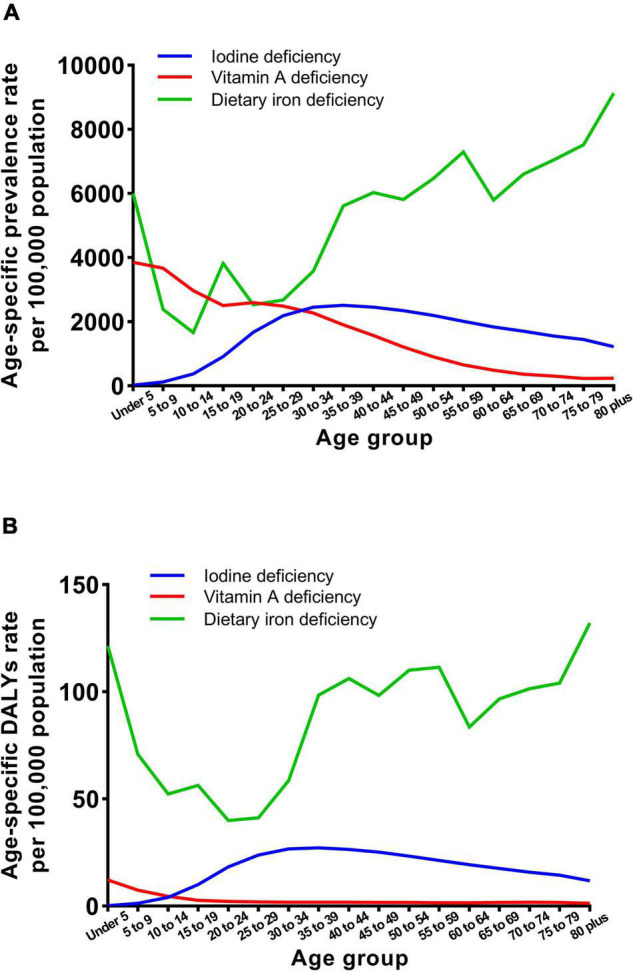
Age-specific prevalence and DALY rates of iodine deficiency, vitamin A deficiency, and dietary iron deficiency in 2019. **(A)** Age-specific prevalence rate. **(B)** Age-specific DALYs rate.

Trends in the sex-specific age-standardized prevalence and DALY rates for micronutrient deficiencies in China from 1990 to 2019 are shown in Figure 1. Generally, the age-standardized prevalence and DALY rates for iodine deficiency, vitamin A deficiency and dietary iron deficiency decreased in both males and females, but the prevalence of iodine deficiency increased in females over the three decades. As shown in Figure 2, the age-specific prevalence rates of dietary iron deficiency increased, but the vitamin A deficiency prevalence decreased with age in 2019. Moreover, the highest iodine deficiency prevalence and DALY rates in 2019 were observed in individuals aged 30–34 years.

The AAPCs in the sex-specific prevalence and DALY rates for micronutrient deficiencies by age group from 1990 to 2019 are presented in Table 1. From 1990 to 2019, the age-standardized prevalence and DALY rates of iodine, vitamin A and dietary iron deficiency in males decreased by 0.6% (95% CI, −0.7 to −0.5%) and 2.0% (95% CI, −2.1 to −1.8%), 6.3% (95% CI, −6.6 to −6.0%) and 3.8% (95% CI, −3.8 to −3.7%), and 3.5% (95% CI, −3.6 to −3.4%) and 4.4% (95% CI, −4.6 to −4.3%), respectively. In females, the age-standardized prevalence rates of vitamin A and dietary iron deficiency decreased by 4.5% (95% CI, −4.8 to −4.2%) and 3.3% (95% CI, −3.4 to −3.2%), respectively, while that of iodine deficiency decreased by 0.8% (95% CI, 0.6 to 1.0%). In addition, a significant increase in the AAPC for iodine deficiency prevalence was observed among females aged 20 years and older.

**TABLE 1:** The average annual percentage changes (AAPCs) in the prevalence of and DALYs due to iodine deficiency, vitamin A deficiency and dietary iron deficiency, 1990–2019.

Age group (year)	AAPC of prevalence,% (95% CI)		AAPC of DALYs,% (95% CI)	
		
	Males	Females	Males	Females
**Iodine deficiency**				
ASR	−0.6 (−0.7 to −0.5)*	0.8 (0.6 to 1.0)*	−2.0 (−2.1 to −1.8)*	−0.2 (−0.4 to 0.0)*
0–4	−2.5 (−2.7 to −2.3)*	−3.2 (−3.4 to −3.1)*	−4.3 (−4.6 to −4.0)*	−5.2 (−5.7 to −4.7)*
5–9	−2.6 (−2.7 to −2.5)*	−3.4 (−3.4 to −3.3)*	−4.6 (−4.9 to −4.3)*	−5.4 (−5.7 to −5.2)*
10–14	−2.3 (−2.4 to −2.2)*	−2.4 (−2.4 to −2.3)*	−4.2 (−4.5 to −3.9)*	−4.2 (−4.6 to −3.9)*
15–19	−1.6 (−1.6 to −1.6)*	−0.6 (−0.7 to −0.5)*	−3.3 (−3.4 to −3.1)*	−2.0 (−2.2 to −1.8)*
20–24	−0.9 (−1.0 to −0.8)*	0.5 (0.4 to 0.6)*	−2.3 (−2.5 to −2.2)*	−0.5 (−0.7 to −0.4)*
25–29	−0.6 (−0.7 to −0.5)*	0.8 (0.6 to 1.0)*	−1.9 (−2.0 to −1.7)*	0.0 (−0.2 to 0.2)
30–34	−0.4 (−0.5 to −0.3)*	1.0 (0.9 to 1.2)*	−1.5 (−1.7 to −1.3)*	0.4 (0.2 to 0.5)*
35–39	−0.3 (−0.4 to −0.1)*	1.1 (0.9 to 1.3)*	−1.4 (−1.6 to −1.3)*	0.4 (0.1 to 0.6)*
40–44	−0.2 (−0.4 to 0.0)*	1.2 (1.0 to 1.3)*	−1.4 (−1.6 to −1.2)*	0.4 (0.2 to 0.6)*
45–49	−0.2 (−0.4 to −0.1)*	1.2 (1.0 to 1.4)*	−1.5 (−1.7 to −1.3)*	0.3 (0.1 to 0.5)*
50–54	−0.2 (−0.4 to −0.1)*	1.2 (1.0 to 1.4)*	−1.5 (−1.7 to −1.4)*	0.3 (0.1 to 0.5)*
55–59	−0.2 (−0.4 to 0.0)*	1.2 (1.0 to 1.4)*	−1.5 (−1.7 to −1.3)*	0.3 (0.1 to 0.5)*
60–64	−0.2 (−0.3 to −0.1)*	1.2 (1.0 to 1.5)*	−1.6 (−1.8 to −1.4)*	0.2 (0.0 to 0.5)
65–69	−0.2 (−0.3 to −0.1)*	1.3 (1.1 to 1.4)*	−1.6 (−1.8 to −1.5)*	0.1 (0.0 to 0.3)
70–74	−0.2 (−0.3 to 0.0)*	1.3 (1.1 to 1.5)*	−1.6 (−1.8 to −1.4)*	0.1 (−0.2 to 0.3)
75–79	0.0 (−0.1 to 0.1)	1.5 (1.3 to 1.7)*	−1.4 (−1.5 to −1.2)*	0.2 (0.0 to 0.5)*
≥80	−0.1 (−0.2 to 0.0)	1.2 (1.0 to 1.4)*	−1.3 (−1.5 to −1.1)*	0.1 (−0.2 to 0.3)
**Vitamin A deficiency**				
ASR	−6.3 (−6.6 to −6.0)*	−4.5 (−4.8 to −4.2)*	−3.8 (−3.8 to −3.7)*	−3.4 (−3.5 to −3.4)*
0–4	−6.2 (−6.5 to −5.9)*	−3.7 (−4.2 to −3.1)*	−4.9 (−5 to −4.7)*	−4.2 (−4.5 to −3.9)*
5–9	−5.1 (−5.4 to −4.7)*	−2.9 (−3.6 to −2.2)*	−4.7 (−4.9 to −4.6)*	−4.3 (−4.4 to −4.2)*
10–14	−5.8 (−6.1 to −5.5)*	−4.6 (−4.8 to −4.4)*	−5.1 (−5.2 to −5.0)*	−5.3 (−5.4 to −5.1)*
15–19	−5.7 (−6.0 to −5.5)*	−4.2 (−4.5 to −4.0)*	0.2 (−0.1 to 0.4)	−0.6 (−0.8 to −0.4)*
20–24	−5.7 (−6.0 to −5.5)*	−4.3 (−4.5 to −4.2)*	−0.2 (−0.5 to 0.1)	−1.0 (−1.3 to −0.7)*
25–29	−5.7 (−6.0 to −5.4)*	−4.1 (−4.3 to −3.9)*	−0.4 (−0.7 to −0.1)*	−1.1 (−1.4 to −0.7)*
30–34	−5.9 (−6.2 to −5.6)*	−3.6 (−3.8 to −3.4)*	−0.4 (−0.6 to −0.3)*	−1.0 (−1.1 to −0.8)*
35–39	−5.8 (−6.1 to −5.5)*	−3.1 (−3.4 to −2.9)*	−0.4 (−0.6 to −0.2)*	−0.7 (−0.9 to −0.6)*
40–44	−5.8 (−6.1 to −5.5)*	−3.3 (−3.6 to −3.0)*	−0.2 (−0.4 to −0.1)*	−0.2 (−0.5 to 0.0)
45–49	−5.9 (−6.1 to −5.6)*	−3.6 (−3.7 to −3.4)*	0.0 (−0.2 to 0.1)	0.2 (−0.1 to 0.5)
50–54	−5.7 (−6.0 to −5.4)*	−4.1 (−4.4 to −3.8)*	0.3 (0.2 to 0.5)*	0.6 (0.4 to 0.8)*
55–59	−5.8 (−6.1 to −5.5)*	−4.5 (−4.7 to −4.3)*	0.6 (0.4 to 0.7)*	0.8 (0.6 to 1.0)*
60–64	−5.8 (−6.1 to −5.5)*	−5.0 (−5.3 to −4.8)*	0.6 (0.4 to 0.8)*	0.7 (0.5 to 1.0)*
65–69	−6.0 (−6.3 to −5.8)*	−5.4 (−5.7 to −5.1)*	0.5 (0.3 to 0.7)*	0.7 (0.4 to 0.9)*
70–74	−6.0 (−6.3 to −5.7)*	−5.8 (−6.1 to −5.6)*	0.4 (0.2 to 0.6)*	0.5 (0.4 to 0.6)*
75–79	−6.0 (−6.2 to −5.7)*	−6.3 (−6.6 to −6.1)*	0.3 (0.2 to 0.4)*	0.3 (0.2 to 0.5)*
≥80	−6.0 (−6.3 to −5.7)*	−6.2 (−6.5 to −5.9)*	0.1 (0.1 to 0.2)*	0.0 (0.0 to 0.1)
**Dietary iron deficiency**				
ASR	−3.5 (−3.6 to −3.4)*	−3.3 (−3.4 to −3.2)*	−4.4 (−4.6 to −4.3)*	−4.5 (−4.6 to −4.4)*
0–4	−3.4 (−3.6 to −3.3)*	−3.5 (−3.7 to −3.3)*	−4.4 (−4.5 to −4.3)*	−4.6 (−4.8 to −4.3)*
5–9	−3.9 (−4.0 to −3.9)*	−5.0 (−5.1 to −4.8)*	−4.4 (−4.5 to −4.3)*	−5.5 (−5.7 to −5.4)*
10–14	−3.8 (−3.9 to −3.7)*	−4.6 (−4.8 to −4.5)*	−4.2 (−4.3 to −4.1)*	−5.2 (−5.3 to −5.0)*
15–19	−2.8 (−2.9 to −2.7)*	−3.9 (−3.9 to −3.8)*	−3.7 (−3.8 to −3.6)*	−5.0 (−5.1 to −4.9)*
20–24	−4.0 (−4.1 to −3.9)*	−3.3 (−3.4 to −3.1)*	−4.7 (−4.8 to −4.6)*	−4.5 (−4.7 to −4.3)*
25–29	−3.8 (−3.9 to −3.7)*	−3.6 (−3.7 to −3.5)*	−4.5 (−4.6 to −4.4)*	−4.9 (−5.0 to −4.7)*
30–34	−3.7 (−3.8 to −3.6)*	−3.1 (−3.4 to −2.8)*	−4.4 (−4.5 to −4.4)*	−4.3 (−4.6 to −4.0)*
35–39	−3.6 (−3.7 to −3.5)*	−2.9 (−3.0 to −2.8)*	−4.4 (−4.5 to −4.3)*	−4.1 (−4.2 to −3.9)*
40–44	−3.7 (−3.8 to −3.6)*	−2.7 (−2.8 to −2.7)*	−4.5 (−4.6 to −4.4)*	−3.9 (−4.0 to −3.8)*
45–49	−3.5 (−3.6 to −3.4)*	−2.6 (−2.7 to −2.5)*	−4.4 (−4.5 to −4.3)*	−3.7 (−3.9 to −3.6)*
50–54	−3.5 (−3.6 to −3.5)*	−2.7 (−2.8 to −2.7)*	−4.5 (−4.5 to −4.4)*	−3.9 (−4.0 to −3.8)*
55–59	−3.2 (−3.3 to −3.1)*	−3.2 (−3.2 to −3.1)*	−4.4 (−4.6 to −4.3)*	−4.4 (−4.5 to −4.3)*
60–64	−3.7 (−3.7 to −3.6)*	−3.5 (−3.5 to −3.4)*	−4.8 (−4.8 to −4.7)*	−4.6 (−4.7 to −4.6)*
65–69	−3.5 (−3.6 to −3.4)*	−3.3 (−3.4 to −3.2)*	−4.7 (−4.8 to −4.6)*	−4.5 (−4.6 to −4.3)*
70–74	−3.4 (−3.6 to −3.3)*	−3.4 (−3.6 to −3.2)*	−4.8 (−4.9 to −4.6)*	−4.6 (−4.8 to −4.4)*
75–79	−3.4 (−3.4 to −3.3)*	−3.6 (−3.6 to −3.5)*	−4.8 (−4.9 to −4.7)*	−4.9 (−5.0 to −4.8)*
≥80	−2.9 (−3.1 to −2.8)*	−3.4 (−3.6 to −3.3)*	−4.6 (−4.8 to −4.4)*	−4.9 (−5.1 to −4.7)*

CI, confidence interval; ASR, age-standardized rate. *Indicates a p-value <0.05.

**FIGURE 3:**
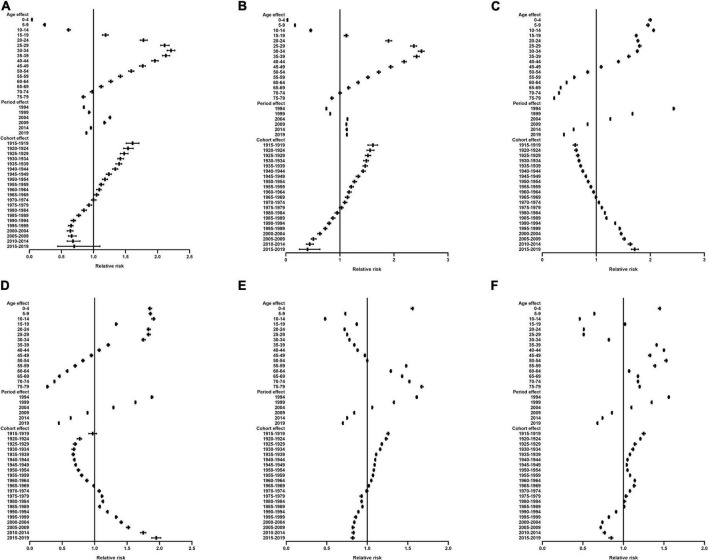
Relative risk of iodine deficiency, vitamin A deficiency and dietary iron deficiency prevalence by sex in China from 1990 to 2019 due to age, period, and cohort effects. **(A)** Iodine deficiency in males. **(B)** Iodine deficiency in females. **(C)** Vitamin A deficiency in males. **(D)** Vitamin A deficiency in females. **(E)** Dietary iron deficiency in males. **(F)** Dietary iron deficiency in females.

The estimated RRs for micronutrient deficiencies due to age, period, and cohort effects are shown in Figure 3 and Supplementary Tables 1–3. When the period and cohort effects were controlled for, significant positive RRs for iodine deficiency were identified in those aged 15–19 years to 65–69 years in both males and females. With regard to the period effect, we observed significant increases in the risk of iodine deficiency from 2004 to 2009 in males and females (Figure 3). Regarding the cohort effect, the RRs for iodine deficiency continuously decreased in later birth cohorts in both males and females (Figure 3). The RRs of vitamin A deficiency associated with net age and period effects significantly decreased with increasing age group and calendar years, respectively; the RRs for vitamin A deficiency continuously increased in later birth cohorts in both males and females (Figure 3). For dietary iron deficiency, significant positive RRs were identified in children aged under 5 years and those aged 55 years and older in both males and females. The RRs for dietary iron deficiency associated with period effects showed significant decreases from 1994 to 2019. Regarding the cohort effect, the RRs for dietary iron deficiency decreased in later birth cohorts in both males and females (Figure 3).

## Discussion

We conducted a broad literature search of multiple databases and found no similar study. The present study is the first comprehensive evaluation of the continuously decreasing trends of the prevalence of and DALYs due to micronutrient deficiencies in China over the past three decades based on GBD data. The standardized methods for estimating the micronutrient deficiency metrics used in the GBD 2019 allowed the comparison of these metrics between years, as well as between sex and age groups. From 1990 to 2019, the age-standardized prevalence and DALY rates for iodine deficiency, vitamin A deficiency and dietary iron deficiency decreased in both males and females, while the prevalence of iodine deficiency increased in females.

Generally, declines in the prevalence and DALYs of iodine deficiency from 1990 to 2019 were observed. This could be explained by the USI program in China, which started in 1996. Over the course of two decades of USI implementation, the Chinese population consecutively experienced an iodine nutrition status of excessive iodine intake for 5 years, more than adequate iodine intake for 10 years, and adequate iodine intake for 5 years (13, 14). According to the latest national survey in 2017, the median urine iodine concentrations in school-age children and adults were 199.75 and 177.89 μg/L, with goiter prevalence rates of 3.50 and 1.17%, respectively, indicating adequate iodine status among the current Chinese population (15). However, national salt monitoring data indicate that adequate consumption of iodized salt has remained below 90% in some developed cities in China, and the low coverage seems to be due to the greater availability of non-iodized salt resulting from concerns that excess iodine might increase the prevalence of thyroid disorders (13). Thus, maintaining the adequately iodized salt consumption rate above 90% is likely to be the main challenge in the next goal of the elimination of iodine deficiency disorders in China.

This study showed that females and young adults were disproportionately affected by a higher prevalence of and more DALYs due to iodine deficiency. This difference may be explained by the fact that androgen stimulates thyroid growth in males, while estrogen has an inhibitory effect in females (16). The high prevalence in females can have a devastating effect on perinatal outcomes, as it can lead to miscarriage, stillbirth, congenital malformations, and neurological defects, and it increases the risks of perinatal and infant mortality. Therefore, the increasing trend of iodine deficiency among females is indeed worthy of attention. The cohort effect on iodine deficiency revealed continuously decreasing trends in the 1915–1919 to 2010–2014 birth cohorts in both males and females. The possible reason is that the later birth cohorts received better education and adequate iodine supplementation and had greater awareness of health and disease prevention than earlier birth cohorts (17). We found that the RR of iodine deficiency increased during 2004–2009, which may be partly due to the fact that national standards for iodized salt were revised to reduce the iodine concentration from 50 to 35 ± 15 mg/kg at the production level in 2002 (14). Therefore, it is necessary to prevent the resurgence of iodine deficiency while also preventing potential harm caused by excess iodine intake.

In this study, an improvement in the prevalence of vitamin A deficiency was observed from 1990 to 2019. This may be the result of the implementation of vitamin A supplementation programs. Large variations were observed among age groups; children aged less than 5 years were the most affected group, consistent with other studies (18–20). Vitamin A status is associated with the growth rate among children (21). This implies that children with a high growth rate have an increased demand for vitamin A. However, a review has suggested that children’s diets in China generally contain only small amounts of plant carotenoids (22). The daily administration of fortified biscuits is suggested for schoolchildren since it has been shown to be effective in reducing vitamin A deficiency and is easily applied in schools (23). Among certain micronutrient deficiencies in China, Vitamin A deficiency seems to be a moderate public health issue. Given that the inadequate intake of vitamin A-rich foods may result in vitamin A deficiency, a comprehensive long-term national strategy needs to be implemented in China for the treatment and prevention of Vitamin A deficiency.

The age-standardized prevalence and DALY rates of dietary iron deficiency in China declined from 1990 to 2019. Dramatic changes in the food supply, dietary behaviors, and national economy in recent years might be possible reasons (24). According to the National Bureau of Statistics of China, the average intake of pork increased from 37.1 g/d in 1992 to 64.33 g/d in 2012 (25). Furthermore, data from the Chinese National Nutrition Survey showed that the total average meat intake increased from 58.9 to 89.7 g/d during 1992–2012 (25). Despite several programs to address iron deficiency in the last three decades, dietary iron deficiency is still one of the most common micronutrient deficiencies in China. When iron intake no longer meets the amount required considering normal iron turnover and loss, iron-deficiency anemia, which accounts for 50% of anemia globally, will occur (26, 27).

The present study showed variation in the burden of dietary iron deficiency by sex; the prevalence and DALY rates in females were higher than those in males. The main reason for the sex difference in the prevalence of iron deficiency is that the iron requirement in women of reproductive age is almost double that in men, and during pregnancy, the amount is triple that in men. When diets are largely plant-based, iron requirements further increase to compensate for the lower bioavailability of iron in plant foods. The impact of female iron deficiency on public health is more pronounced because if measures are not taken in time, iron deficiency will persist in future generations.

Overall, the prevalence of and DALYs due to dietary iron deficiency are higher among children under the age of five and older adults. Young children are at high risk for iron deficiency anemia due to their high dietary iron requirements. In addition, traditional dietary patterns are associated with a high prevalence of iodine deficiency among older Chinese individuals, as previously reported (28). Future research on whether correcting micronutrient deficiencies by promoting an overall healthy diet rather than iron supplementation is an appropriate strategy to prevent anemia in elderly individuals is needed (28). Of note, the definition of dietary iron deficiency used in the current study did not quantify ferritin levels. To better understand the global burden of dietary iron deficiency, it is recommended that the WHO update the data by using gold standard biomarker measurements.

This study analyzed the current conditions and changes in the prevalence of and DALYs due to micronutrient deficiency over the past three decades in China. Our research revealed decreasing burdens of micronutrient deficiencies. However, the interpretation of our study has several limitations. First, bias and gaps in micronutrient deficiency burdens could not be avoided in the modeling process, as described previously (11, 12). Second, there was a lack of high-quality data, particularly regarding the prevalence of other nutritional deficiencies, including zinc, calcium, folate, vitamin B12, and vitamin C deficiencies. Third, due to the absence of mortality data, the DALYs were calculated from only YLDs, which might have led to underestimation. Last, iodine deficiency was estimated using only grade 2 goiter, which might have underestimated the prevalence. Including all forms of iodine deficiency is a goal of future GBD research.

## Conclusion

The burdens of micronutrient deficiencies decreased in China from 1990 to 2019. A higher burden of total micronutrient deficiency was observed in females than in males, and an increasing prevalence of iodine deficiency was observed in females. The results of this study could aid policy makers in evaluating current interventions and guide the future direction of nutritional supplementation, thereby reducing the burden of micronutrient deficiency in China.

## Data Availability Statement

The datasets generated for this study can be found in the GBD at: http://ghdx.healthdata.org/gbd-results-tool.

## Author Contributions

YL conceived the ideas for this research and provided overall guidance. HC, JL, and YL accessed and verified the data. HC and YL prepared the first draft. All authors contributed to the analysis and approved the manuscript.

## Conflict of Interest

The authors declare that the research was conducted in the absence of any commercial or financial relationships that could be construed as a potential conflict of interest.

## Publisher’s Note

All claims expressed in this article are solely those of the authors and do not necessarily represent those of their affiliated organizations, or those of the publisher, the editors and the reviewers. Any product that may be evaluated in this article, or claim that may be made by its manufacturer, is not guaranteed or endorsed by the publisher.

## Footnotes

1
http://ghdx.healthdata.org/gbd-results-tool
