# Supplementary material for: Secular Trends in the Prevalence of and Disability-Adjusted Life Years Due to Common Micronutrient Deficiencies in China From 1990 to 2019: An Age-Period-Cohort Study and Joinpoint Analysis
Source: Front Nutr. 2022 Mar 18;9:754351. doi: 10.3389/fnut.2022.754351 (PMC8971749; doi:10.3389/fnut.2022.754351)
Supplement: Supplementary file 2 [file Table_1.DOC]

Supplementary Table 1. Sex-specific relative risks of iodine deficiency prevalence in China due to age, period, and cohort effects.

|  | Males |  | Females |  |
| --- | --- | --- | --- | --- |
| Factor | RR (95% CI) | P value | RR (95% CI) | P value |
| Age |  |  |  |  |
| 0-4 | 0.04 (0.03 to 0.04) | <0.001 | 0.03 (0.02 to 0.03) | <0.001 |
| 5-9 | 0.24 (0.22 to 0.25) | <0.001 | 0.17 (0.16 to 0.18) | <0.001 |
| 10-14 | 0.61 (0.59 to 0.64) | <0.001 | 0.46 (0.44 to 0.48) | <0.001 |
| 15-19 | 1.19 (1.14 to 1.23) | <0.001 | 1.12 (1.08 to 1.15) | <0.001 |
| 20-24 | 1.78 (1.73 to 1.84) | <0.001 | 1.90 (1.85 to 1.96) | <0.001 |
| 25-29 | 2.11 (2.05 to 2.18) | <0.001 | 2.37 (2.31 to 2.42) | <0.001 |
| 30-34 | 2.21 (2.15 to 2.27) | <0.001 | 2.51 (2.45 to 2.56) | <0.001 |
| 35-39 | 2.13 (2.08 to 2.19) | <0.001 | 2.42 (2.37 to 2.47) | <0.001 |
| 40-44 | 1.96 (1.91 to 2.01) | <0.001 | 2.19 (2.15 to 2.23) | <0.001 |
| 45-49 | 1.77 (1.72 to 1.81) | <0.001 | 1.94 (1.91 to 1.98) | <0.001 |
| 50-54 | 1.59 (1.55 to 1.63) | <0.001 | 1.72 (1.69 to 1.75) | <0.001 |
| 55-59 | 1.42 (1.39 to 1.45) | <0.001 | 1.52 (1.49 to 1.55) | <0.001 |
| 60-64 | 1.27 (1.24 to 1.30) | <0.001 | 1.34 (1.31 to 1.36) | <0.001 |
| 65-69 | 1.12 (1.09 to 1.15) | <0.001 | 1.16 (1.14 to 1.19) | <0.001 |
| 70-74 | 0.98 (0.95 to 1.00) | 0.082 | 1.00 (0.98 to 1.03) | 0.695 |
| 75-79 | 0.84 (0.82 to 0.87) | <0.001 | 0.85 (0.83 to 0.88) | <0.001 |
| Period |  |  |  |  |
| 1994 | 0.85 (0.84 to 0.86) | <0.001 | 0.75 (0.74 to 0.76) | <0.001 |
| 1999 | 0.93 (0.92 to 0.95) | <0.001 | 0.82 (0.82 to 0.83) | <0.001 |
| 2004 | 1.26 (1.24 to 1.27) | <0.001 | 1.14 (1.13 to 1.15) | <0.001 |
| 2009 | 1.17 (1.16 to 1.19) | <0.001 | 1.12 (1.11 to 1.13) | <0.001 |
| 2014 | 0.96 (0.95 to 0.97) | <0.001 | 1.13 (1.11 to 1.14) | <0.001 |
| 2019 | 0.89 (0.88 to 0.90) | <0.001 | 1.13 (1.12 to 1.14) | <0.001 |
| Cohort |  |  |  |  |
| 1915-1919 | 1.61 (1.51 to 1.71) | <0.001 | 1.61 (1.52 to 1.70) | <0.001 |
| 1920-1924 | 1.54 (1.47 to 1.62) | <0.001 | 1.56 (1.50 to 1.63) | <0.001 |
| 1925-1929 | 1.48 (1.42 to 1.54) | <0.001 | 1.52 (1.47 to 1.57) | <0.001 |
| 1930-1934 | 1.42 (1.38 to 1.47) | <0.001 | 1.49 (1.44 to 1.53) | <0.001 |
| 1935-1939 | 1.40 (1.35 to 1.44) | <0.001 | 1.47 (1.43 to 1.51) | <0.001 |
| 1940-1944 | 1.34 (1.30 to 1.38) | <0.001 | 1.43 (1.40 to 1.47) | <0.001 |
| 1945-1949 | 1.24 (1.20 to 1.28) | <0.001 | 1.34 (1.30 to 1.37) | <0.001 |
| 1950-1954 | 1.18 (1.14 to 1.22) | <0.001 | 1.27 (1.24 to 1.31) | <0.001 |
| 1955-1959 | 1.12 (1.09 to 1.16) | <0.001 | 1.21 (1.18 to 1.25) | <0.001 |
| 1960-1964 | 1.09 (1.05 to 1.12) | <0.001 | 1.17 (1.14 to 1.21) | <0.001 |
| 1965-1969 | 1.05 (1.01 to 1.08) | 0.01 | 1.14 (1.10 to 1.17) | <0.001 |
| 1970-1974 | 1.00 (0.97 to 1.04) | 0.924 | 1.09 (1.06 to 1.13) | <0.001 |
| 1975-1979 | 0.93 (0.90 to 0.97) | <0.001 | 1.03 (0.99 to 1.06) | 0.146 |
| 1980-1984 | 0.85 (0.82 to 0.89) | <0.001 | 0.95 (0.91 to 0.98) | 0.004 |
| 1985-1989 | 0.77 (0.74 to 0.80) | <0.001 | 0.87 (0.84 to 0.91) | <0.001 |
| 1990-1994 | 0.69 (0.65 to 0.72) | <0.001 | 0.80 (0.77 to 0.83) | <0.001 |
| 1995-1999 | 0.65 (0.61 to 0.68) | <0.001 | 0.73 (0.70 to 0.76) | <0.001 |
| 2000-2004 | 0.64 (0.59 to 0.68) | <0.001 | 0.63 (0.59 to 0.66) | <0.001 |
| 2005-2009 | 0.66 (0.61 to 0.73) | <0.001 | 0.51 (0.47 to 0.56) | <0.001 |
| 2010-2014 | 0.68 (0.59 to 0.79) | <0.001 | 0.44 (0.38 to 0.51) | <0.001 |
| 2015-2019 | 0.70 (0.44 to 1.10) | 0.118 | 0.40 (0.25 to 0.63) | <0.001 |
| Deviance | 286.00 |  | 374.63 |  |
| AIC | 12.42 |  | 13.89 |  |
| BIC | 30.39 |  | 119.03 |  |

Notes: RR denotes the relative risk of prevalence in a particular age, period, or birth cohort relative to the average level of all age, period, or birth cohort combined. RR, relative risk; CI, confidence interval; AIC, Akaike Information Criterion; BIC, Bayesian Information Criterion.

Supplementary Table 2. Sex-specific relative risks of vitamin A deficiency prevalence in China due to age, period, and cohort effects.

|  | Males |  | Females |  |
| --- | --- | --- | --- | --- |
| Factor | RR (95% CI) | P value | RR (95% CI) | P value |
| Age |  |  |  |  |
| 0-4 | 2.00 (1.97 to 2.02) | <0.001 | 1.85 (1.83 to 1.88) | <0.001 |
| 5-9 | 1.96 (1.93 to 1.98) | <0.001 | 1.86 (1.84 to 1.88) | <0.001 |
| 10-14 | 2.06 (2.04 to 2.08) | <0.001 | 1.91 (1.88 to 1.93) | <0.001 |
| 15-19 | 1.74 (1.72 to 1.76) | <0.001 | 1.33 (1.32 to 1.35) | <0.001 |
| 20-24 | 1.77 (1.75 to 1.79) | <0.001 | 1.83 (1.81 to 1.86) | <0.001 |
| 25-29 | 1.80 (1.78 to 1.83) | <0.001 | 1.83 (1.81 to 1.86) | <0.001 |
| 30-34 | 1.76 (1.74 to 1.78) | <0.001 | 1.75 (1.73 to 1.78) | <0.001 |
| 35-39 | 1.60 (1.58 to 1.63) | <0.001 | 1.21 (1.19 to 1.23) | <0.001 |
| 40-44 | 1.41 (1.39 to 1.43) | <0.001 | 1.07 (1.05 to 1.09) | <0.001 |
| 45-49 | 1.09 (1.07 to 1.11) | <0.001 | 0.95 (0.93 to 0.97) | <0.001 |
| 50-54 | 0.84 (0.82 to 0.86) | <0.001 | 0.82 (0.80 to 0.84) | <0.001 |
| 55-59 | 0.59 (0.58 to 0.61) | <0.001 | 0.70 (0.68 to 0.72) | <0.001 |
| 60-64 | 0.45 (0.44 to 0.46) | <0.001 | 0.58 (0.56 to 0.59) | <0.001 |
| 65-69 | 0.34 (0.33 to 0.35) | <0.001 | 0.46 (0.44 to 0.47) | <0.001 |
| 70-74 | 0.31 (0.30 to 0.32) | <0.001 | 0.38 (0.37 to 0.39) | <0.001 |
| 75-79 | 0.22 (0.21 to 0.23) | <0.001 | 0.27 (0.26 to 0.29) | <0.001 |
| Period |  |  |  |  |
| 1994 | 2.43 (2.42 to 2.45) | <0.001 | 1.88 (1.87 to 1.90) | <0.001 |
| 1999 | 1.67 (1.66 to 1.68) | <0.001 | 1.63 (1.62 to 1.64) | <0.001 |
| 2004 | 1.26 (1.25 to 1.26) | <0.001 | 1.29 (1.28 to 1.30) | <0.001 |
| 2009 | 0.84 (0.83 to 0.84) | <0.001 | 0.89 (0.88 to 0.90) | <0.001 |
| 2014 | 0.58 (0.58 to 0.59) | <0.001 | 0.63 (0.63 to 0.64) | <0.001 |
| 2019 | 0.40 (0.40 to 0.41) | <0.001 | 0.45 (0.44 to 0.45) | <0.001 |
| Cohort |  |  |  |  |
| 1915-1919 | 0.61 (0.57 to 0.65) | <0.001 | 0.97 (0.91 to 1.03) | 0.341 |
| 1920-1924 | 0.63 (0.60 to 0.65) | <0.001 | 0.77 (0.73 to 0.80) | <0.001 |
| 1925-1929 | 0.66 (0.63 to 0.68) | <0.001 | 0.70 (0.67 to 0.72) | <0.001 |
| 1930-1934 | 0.68 (0.66 to 0.70) | <0.001 | 0.68 (0.65 to 0.70) | <0.001 |
| 1935-1939 | 0.71 (0.69 to 0.73) | <0.001 | 0.67 (0.65 to 0.69) | <0.001 |
| 1940-1944 | 0.75 (0.73 to 0.77) | <0.001 | 0.69 (0.67 to 0.70) | <0.001 |
| 1945-1949 | 0.81 (0.79 to 0.82) | <0.001 | 0.71 (0.69 to 0.72) | <0.001 |
| 1950-1954 | 0.85 (0.83 to 0.87) | <0.001 | 0.75 (0.73 to 0.77) | <0.001 |
| 1955-1959 | 0.90 (0.88 to 0.92) | <0.001 | 0.80 (0.78 to 0.82) | <0.001 |
| 1960-1964 | 0.95 (0.93 to 0.97) | <0.001 | 0.88 (0.86 to 0.90) | <0.001 |
| 1965-1969 | 0.99 (0.98 to 1.01) | 0.431 | 0.99 (0.97 to 1.00) | 0.14 |
| 1970-1974 | 1.05 (1.03 to 1.06) | <0.001 | 1.07 (1.05 to 1.09) | <0.001 |
| 1975-1979 | 1.10 (1.09 to 1.12) | <0.001 | 1.11 (1.10 to 1.13) | <0.001 |
| 1980-1984 | 1.16 (1.15 to 1.17) | <0.001 | 1.13 (1.11 to 1.14) | <0.001 |
| 1985-1989 | 1.19 (1.17 to 1.20) | <0.001 | 1.08 (1.06 to 1.09) | <0.001 |
| 1990-1994 | 1.35 (1.34 to 1.36) | <0.001 | 1.20 (1.18 to 1.21) | <0.001 |
| 1995-1999 | 1.43 (1.42 to 1.45) | <0.001 | 1.33 (1.32 to 1.35) | <0.001 |
| 2000-2004 | 1.46 (1.44 to 1.48) | <0.001 | 1.41 (1.39 to 1.43) | <0.001 |
| 2005-2009 | 1.52 (1.49 to 1.54) | <0.001 | 1.52 (1.49 to 1.54) | <0.001 |
| 2010-2014 | 1.63 (1.59 to 1.66) | <0.001 | 1.75 (1.71 to 1.79) | <0.001 |
| 2015-2019 | 1.71 (1.65 to 1.78) | <0.001 | 1.95 (1.88 to 2.02) | <0.001 |
| Deviance | 626.29 |  | 805.29 |  |
| AIC | 17.12 |  | 18.81 |  |
| BIC | 370.69 |  | 549.69 |  |

Notes: RR denotes the relative risk of prevalence in a particular age, period, or birth cohort relative to the average level of all age, period, or birth cohort combined. RR, relative risk; CI, confidence interval; AIC, Akaike Information Criterion; BIC, Bayesian Information Criterion.

Supplementary Table 3. Sex-specific relative risks of dietary iron deficiency prevalence in China due to age, period, and cohort effects.

|  | Males |  | Females |  |
| --- | --- | --- | --- | --- |
| Factor | RR (95% CI) | P value | RR (95% CI) | P value |
| Age |  |  |  |  |
| 0-4 | 1.56 (1.54 to 1.57) | <0.001 | 1.45 (1.43 to 1.46) | <0.001 |
| 5-9 | 0.73 (0.72 to 0.73) | <0.001 | 0.64 (0.64 to 0.65) | <0.001 |
| 10-14 | 0.48 (0.47 to 0.49) | <0.001 | 0.46 (0.46 to 0.47) | <0.001 |
| 15-19 | 0.87 (0.86 to 0.88) | <0.001 | 1.02 (1.01 to 1.03) | 0.001 |
| 20-24 | 0.72 (0.71 to 0.73) | <0.001 | 0.51 (0.51 to 0.52) | <0.001 |
| 25-29 | 0.75 (0.75 to 0.76) | <0.001 | 0.51 (0.50 to 0.52) | <0.001 |
| 30-34 | 0.78 (0.77 to 0.79) | <0.001 | 0.82 (0.81 to 0.83) | <0.001 |
| 35-39 | 0.84 (0.83 to 0.85) | <0.001 | 1.41 (1.40 to 1.42) | <0.001 |
| 40-44 | 0.88 (0.87 to 0.89) | <0.001 | 1.50 (1.49 to 1.51) | <0.001 |
| 45-49 | 0.97 (0.96 to 0.98) | <0.001 | 1.33 (1.31 to 1.34) | <0.001 |
| 50-54 | 1.00 (0.99 to 1.01) | 0.484 | 1.53 (1.51 to 1.54) | <0.001 |
| 55-59 | 1.48 (1.47 to 1.49) | <0.001 | 1.39 (1.37 to 1.40) | <0.001 |
| 60-64 | 1.29 (1.28 to 1.30) | <0.001 | 1.07 (1.06 to 1.08) | <0.001 |
| 65-69 | 1.43 (1.42 to 1.44) | <0.001 | 1.18 (1.17 to 1.19) | <0.001 |
| 70-74 | 1.52 (1.51 to 1.53) | <0.001 | 1.18 (1.17 to 1.19) | <0.001 |
| 75-79 | 1.67 (1.66 to 1.69) | <0.001 | 1.20 (1.19 to 1.21) | <0.001 |
| Period |  |  |  |  |
| 1994 | 1.61 (1.60 to 1.62) | <0.001 | 1.56 (1.55 to 1.57) | <0.001 |
| 1999 | 1.33 (1.32 to 1.33) | <0.001 | 1.35 (1.34 to 1.35) | <0.001 |
| 2004 | 1.06 (1.06 to 1.07) | <0.001 | 1.10 (1.09 to 1.10) | <0.001 |
| 2009 | 0.84 (0.83 to 0.84) | <0.001 | 0.86 (0.85 to 0.86) | <0.001 |
| 2014 | 0.75 (0.75 to 0.76) | <0.001 | 0.74 (0.74 to 0.75) | <0.001 |
| 2019 | 0.70 (0.69 to 0.70) | <0.001 | 0.68 (0.67 to 0.68) | <0.001 |
| Cohort |  |  |  |  |
| 1915-1919 | 1.26 (1.24 to 1.27) | <0.001 | 1.25 (1.23 to 1.27) | <0.001 |
| 1920-1924 | 1.23 (1.22 to 1.25) | <0.001 | 1.21 (1.20 to 1.22) | <0.001 |
| 1925-1929 | 1.18 (1.17 to 1.19) | <0.001 | 1.14 (1.13 to 1.16) | <0.001 |
| 1930-1934 | 1.16 (1.15 to 1.17) | <0.001 | 1.12 (1.10 to 1.13) | <0.001 |
| 1935-1939 | 1.11 (1.10 to 1.12) | <0.001 | 1.08 (1.07 to 1.09) | <0.001 |
| 1940-1944 | 1.10 (1.09 to 1.10) | <0.001 | 1.05 (1.04 to 1.06) | <0.001 |
| 1945-1949 | 1.09 (1.08 to 1.10) | <0.001 | 1.04 (1.04 to 1.05) | <0.001 |
| 1950-1954 | 1.08 (1.07 to 1.09) | <0.001 | 1.05 (1.04 to 1.06) | <0.001 |
| 1955-1959 | 1.07 (1.06 to 1.08) | <0.001 | 1.08 (1.08 to 1.09) | <0.001 |
| 1960-1964 | 1.05 (1.04 to 1.06) | <0.001 | 1.14 (1.13 to 1.15) | <0.001 |
| 1965-1969 | 1.02 (1.01 to 1.03) | <0.001 | 1.13 (1.12 to 1.15) | <0.001 |
| 1970-1974 | 0.99 (0.98 to 1.01) | 0.327 | 1.08 (1.07 to 1.09) | <0.001 |
| 1975-1979 | 0.93 (0.91 to 0.94) | <0.001 | 1.03 (1.02 to 1.05) | <0.001 |
| 1980-1984 | 0.93 (0.92 to 0.94) | <0.001 | 1.01 (1.00 to 1.03) | 0.017 |
| 1985-1989 | 0.94 (0.93 to 0.95) | <0.001 | 1.01 (1.00 to 1.02) | 0.078 |
| 1990-1994 | 0.89 (0.88 to 0.90) | <0.001 | 0.91 (0.90 to 0.92) | <0.001 |
| 1995-1999 | 0.86 (0.84 to 0.87) | <0.001 | 0.82 (0.81 to 0.83) | <0.001 |
| 2000-2004 | 0.84 (0.83 to 0.85) | <0.001 | 0.74 (0.73 to 0.75) | <0.001 |
| 2005-2009 | 0.82 (0.81 to 0.84) | <0.001 | 0.72 (0.71 to 0.73) | <0.001 |
| 2010-2014 | 0.83 (0.81 to 0.84) | <0.001 | 0.77 (0.75 to 0.78) | <0.001 |
| 2015-2019 | 0.82 (0.80 to 0.84) | <0.001 | 0.85 (0.82 to 0.87) | <0.001 |
| Deviance | 519.94 |  | 1124.40 |  |
| AIC | 16.88 |  | 23.30 |  |
| BIC | 264.33 |  | 868.79 |  |

Notes: RR denotes the relative risk of prevalence in a particular age, period, or birth cohort relative to the average level of all age, period, or birth cohort combined. RR, relative risk; CI, confidence interval; AIC, Akaike Information Criterion; BIC, Bayesian Information Criterion.
